# Mobilized Adult Pituitary Stem Cells Contribute to Endocrine Regeneration in Response to Physiological Demand

**DOI:** 10.1016/j.stem.2013.07.006

**Published:** 2013-10-03

**Authors:** Karine Rizzoti, Haruhiko Akiyama, Robin Lovell-Badge

**Affiliations:** 1 Division of Stem Cell Biology and Developmental Genetics, MRC National Institute for Medical Research, London NW7 1AA, UK; 2 Department of Orthopaedic Surgery, Kyoto University Graduate School of Medicine, Kyoto 606-8507, Japan

## Abstract

Pituitary hormone deficiencies, with Growth Hormone deficiency being most frequent (1 in 3,500-10,000 births), cause significant morbidity. Regeneration of missing endocrine cells would be a significant improvement over hormone replacement therapies, which incur side effects and do not mimic physiological secretion patterns. Recent in vitro studies have identified a population of adult pituitary progenitors that express the HMG box transcription factors SOX2 and SOX9. Here, we apply cell-lineage tracing analysis to demonstrate that SOX2- and SOX9-expressing progenitors can self-renew and give rise to endocrine cells in vivo, suggesting that they are tissue stem cells. Moreover, we show that they can become mobilized and differentiate into the appropriate endocrine cell types in response to physiological stress. Our results highlight the pituitary as a model for exploring how physiological changes influence stem cell behavior and suggest that manipulation of endogenous pituitary stem cells is a potential therapeutic strategy for pituitary deficiencies.

**Video Abstract:**

## Highlights

• SOX2 and SOX9 are expressed in embryonic and adult pituitary progenitors
• SOX2- and SOX9-positive progenitors self-renew and generate endocrine cells in vivo
• In the adult, these tissue stem cells mostly self-renew
• Physiological demand can activate these cells and induce their differentiation

## Graphical Abstract

**Figure:**
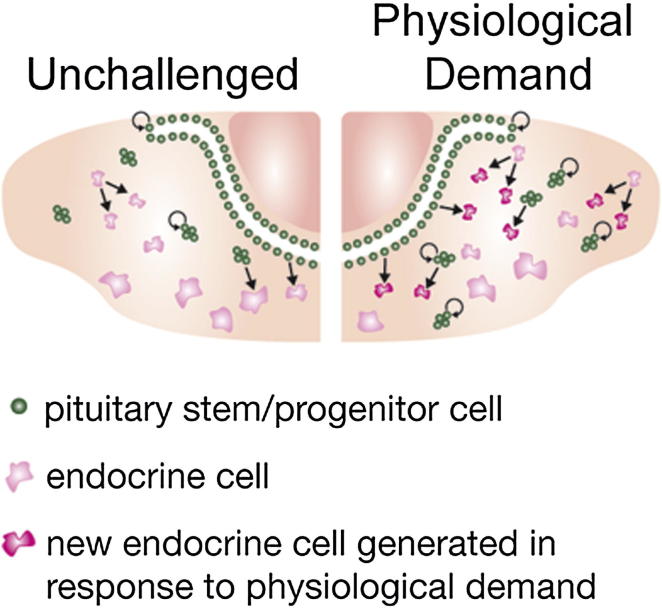

## Teaser

A stem cell population present in the adult pituitary can be mobilized in response to physiological demand to make new pituitary tissue.

## Introduction

Throughout the life of an animal, organ-specific differentiated cell types are renewed. This can be achieved by cell division, as shown by hepatocytes in the liver, and/or by differentiation from a pool of undifferentiated multipotent progenitors or stem cells, such as the gastrointestinal epithelium. While adult progenitors or stem cells are being found in an increasing number of organs, their participation in tissue homeostasis varies according to rates of cell turnover and the ability of differentiated cell types to self-renew. One central goal of regenerative medicine is to be able to instruct resident tissue stem cells to repair a deficient organ. It is therefore crucial to know how organ-specific tissue stem cells function under normal physiological situations and also whether it is possible to stimulate their regenerative potential.

The pituitary is an endocrine gland involved in maintaining body homeostasis and controlling physiological processes such as reproductive maturation and function. It does so by secreting hormones under control of the hypothalamus, which acts to centralize peripheral information. The pituitary is an organ with a low cell turnover where differentiated endocrine cells are able to divide, but they do so rarely (Levy, 2002). Physiological situations evolve during life: growth is crucial in juvenile animals, later, sexual maturation occurs, and, in the female, pregnancy and lactation can take place. All these processes are controlled by specific hormonal outputs from the pituitary, which must constantly modulate its hormonal secretions appropriately. This adaptability can be achieved in different ways, such as by modifying levels and/or temporal patterns of hormonal synthesis and secretion, but it has also been proposed that a population of adult progenitor/stem cells is involved. Recently, several labs have characterized (likely overlapping) populations of such cells (for review see Rizzoti, 2010). The ability to control the activity of these progenitors in vivo, or to use them in regenerative or cell transplant therapies, could be used to manipulate physiological states or to treat congenital or acquired pituitary hormone deficiencies, which are associated with significant morbidity. This could also alleviate both the side-effects and cost of current hormone replacement and substitution therapies (for review see Castinetti et al., 2011).

We showed that pituitary progenitors express the SRY-related HMG box transcription factors SOX2 and SOX9 (Fauquier et al., 2008). These belong to different subfamilies, SOXB1 and SOXE, and are therefore likely to regulate different sets of target genes. Both proteins have pleiotropic roles during organogenesis but also in different adult stem cell populations (for review see Sarkar and Hochedlinger, 2013). During pituitary development, SOX2 is expressed initially in all cells of Rathke’s Pouch (RP), an outpocketing of the oral ectoderm that gives rise to the anterior and intermediate pituitary (Fauquier et al., 2008), where it is required for progenitor proliferation (Jayakody et al., 2012 and our unpublished data), while SOX9 is expressed later, after the first and main wave of embryonic progenitor cell cycle exit (Davis et al., 2011; Fauquier et al., 2008). Expression of both proteins is downregulated upon endocrine differentiation. This pattern is still present in the adult where SOX2;SOX9-double-positive cells form the epithelium lining the pituitary cleft, the remnant of RP lumen, proposed as a pituitary stem cell niche, and are also scattered in the pituitary parenchyma (for review see Rizzoti, 2010). In vitro, pituitary spheres initially express SOX2, then SOX9, and can give rise to endocrine cells (Fauquier et al., 2008). In vivo, it has been suggested that SOX2^+ve^ cells could generate new endocrine cells in experimental endocrine cell elimination models, where limited regeneration was observed (Fu et al., 2012; Fu and Vankelecom, 2012), although these studies were correlative and did not involve fate-mapping.

In this study, we demonstrate that SOX2- and SOX9-positive progenitors are the sphere-forming cells in vitro and, from lineage analysis, demonstrate that they give rise to endocrine cells in vivo, both in the embryo and postnatally. Long-term maintenance of progenitor identity suggests that SOX2- and SOX9-expressing cells are tissue stem cells, although these give rise to differentiated progeny infrequently in unchallenged adult animals. However, in situations where the pituitary is known to show plasticity, we show that adult pituitary stem cells can make a significant contribution to the pool of new endocrine cells generated in response to physiological demand. This represents a first step toward the use of pituitary stem cells to modulate endocrine output and treat deficits.

## Results

### SOX2- and SOX9-Positive Cells Are the Pituisphere-Forming Cells

**Figure 1:**
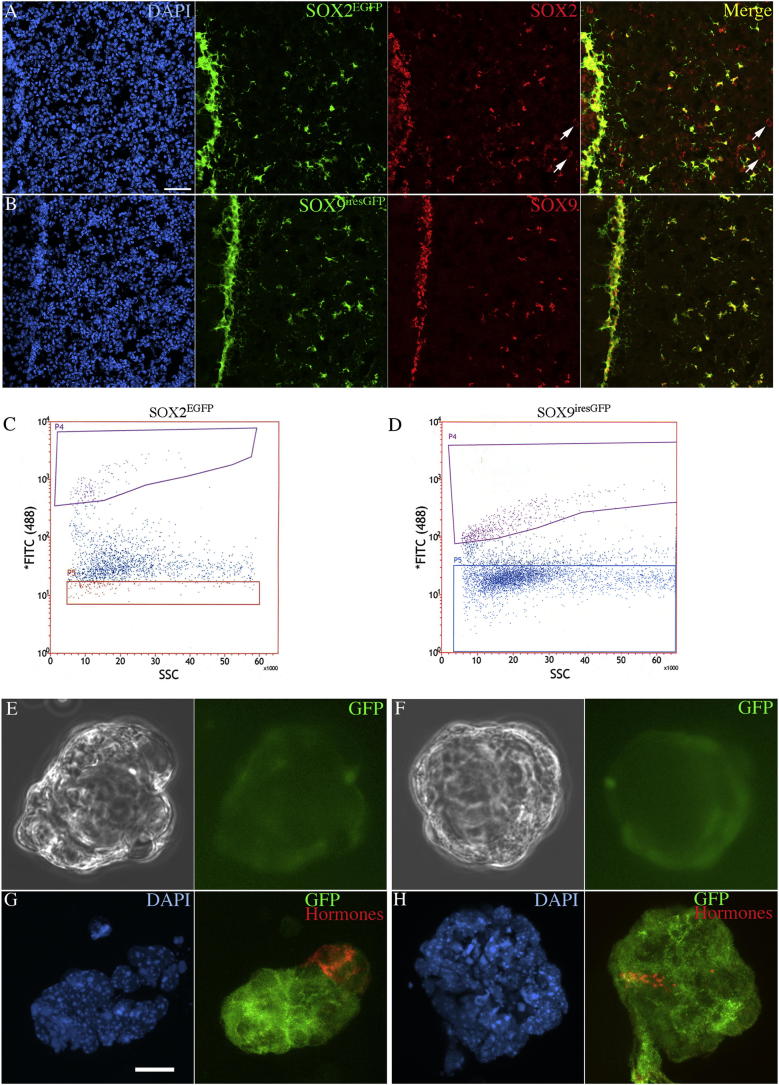
SOX2- and SOX9-Positive Cells Are the Sphere-Forming Cells (A and B) SOX2-EGFP and SOX9-IRES-GFP alleles faithfully recapitulate SOX2 (A) and SOX9 (B) expression in the pituitary. Note that SOX2-EGFP is not present in cells where SOX2 staining is cytoplasmic (arrows). (C and D) FAC sorting of 3 week-old *Sox2*^*EGFP/+*^ and 4-week-old *Sox9*^*iresGFP/iresGFP*^. (E and F) Spheres are observed in SOX2-EGFP and SOX9-IRES-GFP-positive fractions (bright field and live fluorescence). (G and H) Spheres obtained from SOX2-positive cells (G, DAPI and immunofluorescence for GFP and anterior pituitary hormones) and SOX9-positive cells (H, DAPI and immunofluorescence for GFP and all anterior pituitary hormones) are able to give rise to hormonal cells in differentiating conditions. Scale bars: 50 μm for (A) and (B) and 5 μm for (G) and (H). See also Figure S1.

We initially checked that the targeted alleles *Sox2*^*EGFP*^ and *Sox9*^*iresGFP*^ recapitulated SOX2 and SOX9 expression patterns, respectively, in the pituitary (Figures 1A and 1B). We isolated SOX2-positive cells by FACS after dissociation of pituitaries from *Sox2*^*EGFP/+*^ mice (Ellis et al., 2004) (Figure 1C). We plated the EGFP-positive fraction and, in parallel, an equivalent number of pituitary EGFP-negative cells in sphere forming conditions. After 1 week in culture, only the EGFP-positive, SOX2-positive fraction contained pituispheres from which hormone-positive endocrine cells could be further differentiated (Figure 1G) (Andoniadou et al., 2012). We also isolated SOX9-positive cells using *Sox9*^*iresGFP/iresGFP*^ mice (Figure 1D) (Furuyama et al., 2011). Upon being plated, the GFP-positive fraction contained pituispheres that gave rise to endocrine cells (Figure 1F). A very small number of GFP^+ve^ spheres was observed when the entire GFP-negative fraction was plated from either *Sox2*^*EGFP/+*^ or *Sox9*^*iresGFP/iresGFP*^ mice (0.0035%, n = 2). It is most likely that these originate from cells that were not sorted appropriately. However, as we proposed previously, the spheres in the latter could come from SOX2^+ve^;SOX9^−ve^ cells (Fauquier et al., 2008). Similarly the spheres in the SOX2-EGFP^−ve^ fraction could come from SOX9^+ve^;SOX2^−ve^ cells (Figure S1 available online). Our previous results (Fauquier et al., 2008) showed that SOX9 only appeared after a few days in sphere cultures. This is in apparent contrast with the sphere-forming ability of the SOX9-IRES-GFP-positive fraction. We therefore further examined spheres derived from the latter, finding that SOX9 was downregulated, while GFP persists during early sphere formation, only to resume after 6–7 days (Figure S1). In conclusion, both SOX2- and SOX9-positive cells from the adult pituitary have sphere-forming ability, but during their initial formation the expression of the two proteins appears to recapitulate their embryonic pattern.

### SOX2- and SOX9-Positive Cells Generate Endocrine Cells in the Embryonic Pituitary

During embryogenesis, SOX2 is expressed throughout RP at least from 10.5 days post coitum (dpc), while SOX9 starts to be faintly expressed in a few cells at 12.5 dpc and is then upregulated from 14.5 dpc until adulthood, when both proteins clearly colocalize (Figure S2 and Fauquier et al., 2008). In order to investigate the differentiation potential of these progenitors in vivo, we used *Sox2*^*CreERT2*^ (Arnold et al., 2011) and *Sox9*^*ires-CreERT2*^ alleles (Furuyama et al., 2011). These were bred into mice carrying the Cre-reporter *R26R*^*EYFP*^. Cre activity was induced with 4-hydroxy-tamoxifen (4-OHT) in embryos at several stages and pituitaries harvested at 18.5 dpc, toward the end of gestation.

**Figure 2:**
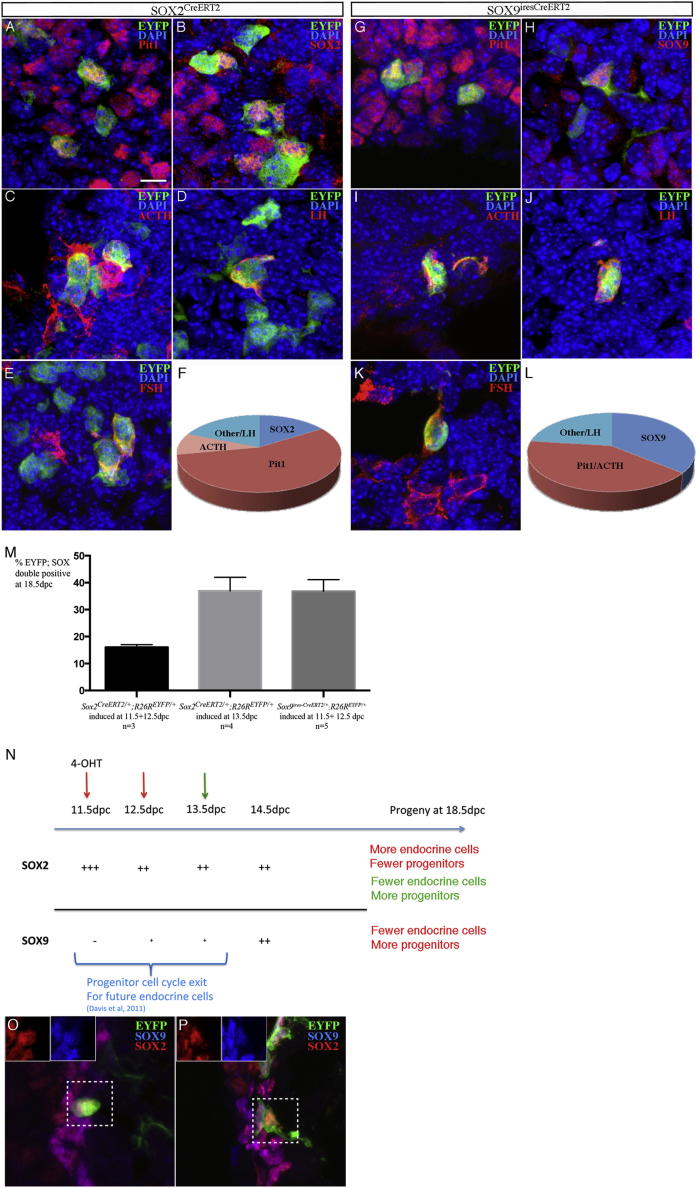
SOX2- and SOX9-Positive Progenitors Give Rise to Endocrine Cells in the Embryo and to SOX2;SOX9-Double-Positive Putative Progenitors in the Adult (A–F) *Sox2*^*CreERT2*^ embryonic lineage tracing. (G–L) *Sox9*^*ires-CreERT2*^ embryonic lineage tracing. 4-OHT (0.1 to 0.15 mg/g/day) was given to pregnant females at 11 and 12.5 dpc. Pituitaries were harvested at 18.5 dpc and immunofluorescence was performed for GFP, SOX2, SOX9, and anterior pituitary cell lineages (Pit-1 for GH, PRL, and TSH-secreting cells and their progenitors, ACTH for corticotrophs, and LH and FSH for gonadotrophs). (L) Double-positive cells were counted for each lineage in at least three embryos. “Other” refers to the proportion of EYFP^+ve^ cells that were not identified. (M) Proportions of SOX^+ve^ progenitors stained at 18.5 dpc after induction of *Sox2*^*CreERT2*^ early, before SOX9 is expressed (11.5+12.5), or late, as SOX9 is upregulated (13.5 dpc), along with *Sox9*^*ires-CreERT2*^ results. Data are presented as mean ± SEM. (N) Model recapitulating results from the embryonic lineage tracings according to expression timing of SOX2, SOX9, and Cre induction. (O and P) Fetal SOX2- (O) and SOX9- (P) positive progenitors give rise to adult progenitors. 4-OHT (0.1 to 0.15 mg/g) was given to pregnant females at 11.5 (*Sox9*^*ires-CreERT2*^) and 13.5 dpc (*Sox2*^*CreERT2*^). Pituitaries were harvested postnatally at 8 weeks. EYFP expression was observed in particular in SOX2/9-double-positive progenitors. Insets show SOX2 and SOX9 staining in triple-positive cells. Scale bars: 10 μm for all panels. See also Table S1.

When we induce Cre activity at 9.5 and 10.5 dpc or 11.5 and 12.5 dpc in *Sox2*^*CreERT2/+*^*;R26R*^*EYFP/+*^ embryos, before or at the start of endocrine differentiation (Davis et al., 2011), we observe EYFP-positive cells in all endocrine lineages at the end of gestation (Figures 2A–2F). More precisely, when induction is performed at 11.5 and 12.5 dpc, 56% of EYFP^+ve^ cells in the anterior lobe (excluding those in the cleft) are Pit-1 positive (representing somatrotrophs, lactotrophs, and thyrotrophs and their immediate progenitors), while 9% are adrenocorticotropic hormone (ACTH) positive corticotrophs (Figure 2F, Table 1, and Table S1). We also observe luteinizing hormone (LH)- and follicle-stimulating hormone (FSH)-positive gonadotrophs within the SOX2 lineage, but because gonadotroph repartition is not homogenous in the gland (Budry et al., 2011), we cannot count the proportion of EYFP^+ve^ cells that become gonadotrophs. Some EYFP^+ve^ cells express SOX2 (16%, in the anterior lobe); moreover, the cleft also contains a significant proportion of EYFP^+ve^ cells (but note that these are not included in the quantitative analysis). These cells are all hormone-negative, showing that not all progenitors differentiate and/or reflecting symmetric or asymmetric self-renewal divisions (Figure 2F). About 20% of EYFP-positive cells did not costain with Pit-1, ACTH, or SOX2. These are likely to be differentiating progenitors (for the gonadotroph and corticotroph lineages), gonadotrophs, and probably Pit-1-independent thyrotrophs.

We next used the *Sox9*^*ires-CreERT2/+*^ animals and noticed an obvious reduction in recombination efficiency compared to *Sox2*^*CreERT2*^ (Figure S2). While it is clear that there are many more SOX2- than SOX9-positive cells at early stages (12.5 dpc), even when induction is performed later (13.5 dpc), when both proteins are coexpressed, or in the adult (see below), Sox2-CreERT2 is more efficient. This may reflect low expression levels of SOX9 compared to SOX2 in the pituitary or a difference in expression or activity of the CreERT2 cassette. Moreover, because CreERT2 replaces the SOX2 open reading frame (ORF) in *Sox2*^*CreERT2/+*^ animals (Arnold et al., 2011), one copy of *Sox2* is missing. This is known to affect pituitary development and function (Kelberman et al., 2006). Although the phenotype is mild and affects all hormonal cell types, this may also account for some quantitative differences seen between the two Cre drivers. In contrast, CreERT2 was inserted following an IRES into the *Sox9* 3′UTR because SOX9 heterozygosity is lethal (Soeda et al., 2010). Translation following an IRES is often less efficient compared to that initiated from the endogenous transcript. Nevertheless, the lower recombination efficiency is beneficial for clonal analysis and SOX9-IRES-CreERT2 appears to be an ideal tool to perform lineage tracing in the pituitary.

When we induce Cre activity in *Sox9*^*ires-CreERT2/+*^*;R26R*^*EYFP/+*^ embryos at 11.5 and 12.5 dpc, we also observe EYFP-positive cells in all lineages (Figures 2G–2L, Table 1, and Table S1). More precisely, we mainly observe EYFP-positive cells in the Pit-1 lineage and in some rare corticotrophs (together representing 41% of the EYFP^+ve^ cells). We very rarely observe EYFP^+ve^ gonadotrophs. This is likely explained by both the lower recombination efficiency and the fact that LH- and FSH-positive cells represent a relatively small population. Finally, a significant percentage of EYFP^+ve^ cells still express SOX9 at 18.5 dpc (36%). Again a proportion of cells (about 20%) are not costained by Pit-1, ACTH, or SOX9.

Comparing the two lineage tracing methods, there is a significant reduction in differentiated progeny when using *Sox9* rather than *Sox2* to drive *CreERT2* (41.4% versus 64.7% Pit-1/ACTH cells) and a corresponding increase in progenitors (36.8% versus 16%, respectively). It is very likely that EYFP^+ve^ cells observed with the former are induced later than with the latter, as SOX2 is already strongly expressed at 11.5 dpc, while SOX9 expression only becomes significant at 14.5 dpc. Progenitor cell cycle exit in the pituitary has been shown to occur for all endocrine populations mainly between 11.5 and 13.5 dpc (Davis et al., 2011). Therefore lineage tracing performed at the end, and/or after this phase, as would happen with *Sox9*^*ires-CreERT2*^, should give rise to fewer differentiated progeny, and, in consequence, increased retention of the lineage marker in progenitors. To verify this hypothesis, we gave 4-OHT to *Sox2*^*CreERT2/+*^*;R26R*^*EYFP/+*^ embryos at 13.5 dpc, and observed a higher percentage of EYFP;SOX2 double-positive cells (37%, Figure 2M, Table 1, and Table S1), comparable to that obtained with *Sox9*^*ires-CreERT2*^.

Taken together, these results show that SOX2- and SOX9-positive precursors differentiate into endocrine cells in the developing pituitary, and moreover, that they are in agreement with birth-dating data showing that progenitors differentiate more efficiently before 13.5 dpc (Davis et al., 2011). When Cre activity is induced in *Sox2*^*CreERT2/+*^*;R26R*^*EYFP/+*^ embryos before 13.5 dpc, at a time prior to the onset of SOX9 expression, a greater proportion of progenitors differentiate compared to its later induction, after SOX9 is upregulated and when Cre activity can first be induced in *Sox9*^*ires-CreERT2/+*^*;R26R*^*EYFP/+*^embryos (Figure 2N).

### Embryonic SOX2 and SOX9-Positive Cells Give Rise to Adult Progenitors

It has been proposed that embryonic and adult progenitors derive from distinct cell populations, because lineage tracing using a NestinCre transgene only gave rise to significant endocrine progeny postnatally (Gleiberman et al., 2008). To test this hypothesis, we induced Cre activity in *Sox2*^*CreERT2/+*^*;R26R*^*EYFP/+*^ (13.5 dpc) and *Sox9*^*ires-CreERT2/+*^*;R26R*^*EYFP/+*^ (11.5 dpc) embryos and harvested pituitaries from newborns and adults. Most of the labeled cell progeny had differentiated (data not shown), but we were able to detect, in both sets of samples, EYFP;SOX2;SOX9-triple-positive cells (Figures 2K and 2L). More precisely, in both *Sox2*^*CreERT2/+*^*;R26R*^*EYFP/+*^ (n = 2, SD = 1.5; 8 and 17 weeks) and *Sox9*^*ires-CreERT2/+*^*;R26R*^*EYFP/+*^ (n = 3, SD = 2.5; 8 to 26 weeks) pituitaries, an average of 3% of EYFP-positive cells remained SOX2- and SOX9-positive, respectively, in the anterior lobe (excluding the cleft). Therefore, in agreement with recent data for other SOX2^+ve^ progenitors in various adult epithelial tissues (Arnold et al., 2011), a proportion of embryonic SOX2;SOX9-positive progenitors remain undifferentiated and maintain their identity until adulthood.

### SOX2- and SOX9-Positive Cells Give Rise to Endocrine Cells during the Postnatal Pituitary Growth Phase

**Figure 3:**
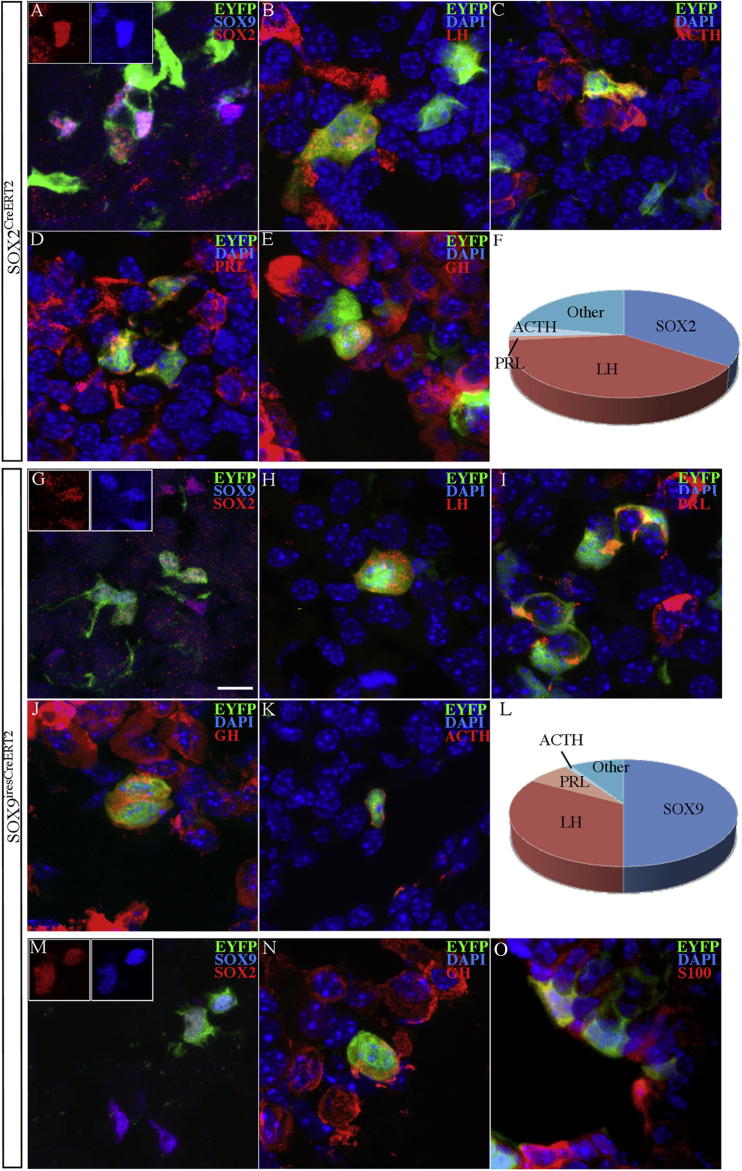
SOX2- and SOX9-Positive Postnatal Progenitors Give Rise to Endocrine Cells and Folliculo-Stellate Cells (A–F) *Sox2*^*CreERT2*^ postnatal lineage tracing. (G–L) *Sox9*^*ires-CreERT2*^ postnatal lineage tracing. 4-OHT (1 mg) was given to P0–P2 pups and pituitaries were harvested at 4 weeks. (A–L) Immunofluorescence for EYFP, SOX2, SOX9, and anterior pituitary hormones were then performed. Double-positive cells were counted for each lineage in at least four animals. “Other” refers to the proportion of EYFP^+ve^ cells that were not identified. SOX2 and SOX9 progenitors participate in pituitary growth postnatally and give rise to differentiated cells in all endocrine lineages. (M–O) *Sox9*^*ires-CreERT2*^ adult lineage tracing. Tamoxifen (5 mg/25 g/day for 3 days) was given to 8 week-old animals. Pituitaries were harvested at one year (M and N) or after 2 months (O). (M) Triple SOX2;SOX9;EYFP and double (N) GH;EYFP and (O) S100;EYFP are shown. Insets show SOX2 and SOX9 staining in triple-positive cells. Scale bars: 10 μm for all panels. See also Figure S3 and Table S1.

The murine pituitary is characterized by a phase of rapid growth postnatally to reach its adult size. Differentiated endocrine cells are known to proliferate during this phase (Kominami et al., 2003). We decided to study the contribution of SOX2^+ve^ and SOX9^+ve^ cells during this period by inducing Cre activity in newborns and analyzing pituitaries at 4 weeks (Figure 3). As observed in embryos, recombination is much less efficient in *Sox9*^*ires-CreERT2/+*^*;R26R*^*EYFP/+*^ versus *Sox2*^*CreERT2/+*^*;R26R*^*EYFP/+*^ animals. While we routinely counted more than 200 EYFP^+ve^ cells/animal/hormone for *Sox2*^*CreERT2/+*^*;R26R*^*EYFP/+*^ samples, for *Sox9*^*ires-CreERT2/+*^*;R26R*^*EYFP/+*^ we typically were only able to count 50 to 100 cells (Table 1 and Table S1).

Using *Sox2*^*CreERT2*^, we mainly observed colocalization of EYFP with LH^+ve^ (and FSH, data not shown) gonadotrophs (39%, Table 1). ACTH^+ve^;EYFP^+ve^ cells were detected at a lower frequency (3.2%), and while EYFP^+ve^ lactotrophs, somatotrophs, and thyrotrophs were present, they were rare (<1%). A large proportion of EYFP^+ve^ cells were SOX2^+ve^ (35%), suggesting that they were the products of self-renewal divisions (Figures 3A–3F). In 8-week-old animals, a similar percentage of EYFP;LH- and EYFP;ACTH-double-positive cells are still present (Table 1 and Table S1). A proportion of EYFP^+ve^ cells (about 20%, as estimated from double staining, and confirmed in one animal by triple EYFP;SOX2;ACTH-LH staining, data not shown) appears to be negative for both SOX2 and LH;ACTH. These cells comprise a small percentage of prolactin (PRL)-, thyroid-stimulating hormone (TSH)-, and growth hormone (GH)-positive cells as well as others that are presumably differentiating and not yet expressing hormones.

Using *Sox9*^*ires-CreERT2*^, we also detected EYFP mainly in LH^+ve^ (and FSH^+ve^) gonadotrophs (34%) (Table 1, Table S1, and data not shown) and in some lactotrophs. In one animal where recombination was particularly efficient, we were able to detect a few GH^+ve^;EYFP^+ve^ and ACTH^+ve^;EYFP^+ve^ cells. A significant percentage of the EYFP^+ve^ cells still expressed SOX9 (50%), higher than that observed with *Sox2*^*CreERT2*^ (Figures 3G–3L). The percentage of EYFP^+ve^ gonadotrophs remained high in 8-week-old animals (24%; n = 2, SD = 9.8, 1 female and 1 male). A proportion of EYFP^+ve^ cells were not identified, but these were less frequent compared to those seen in *Sox2*^*CreERT2*^ lineage tracing experiments. With triple staining (EYFP;SOX9;LH-PRL, n = 3), about 10% of EYFP^+ve^ cells were not identified (Figure 3L).

We suspected that the prominent differentiation toward gonadotroph fate at these stages could be due to the estrogen (E2) receptor modulator tamoxifen. We therefore counted gonadotrophs in tamoxifen- versus oil-treated animals and observed many more in the former (Figure S3), which provides an explanation for the high percentage of LH-positive cells in the progeny of both SOX2- and SOX9-positive progenitors.

In summary, SOX2^+ve^ and SOX9^+ve^ progenitors can differentiate into all endocrine cell lineages postnatally. Despite the fact that more than 95% of progenitors are SOX2;SOX9-double-positive (Figure S1), there are two main differences between *Sox2*^*CreERT2*^ and *Sox9*^*ires-CreERT2*^ lineage tracing experiments. First, a greater proportion of descendants appear to lose progenitor marker expression with *Sox2*^*CreERT2*^ compared to *Sox9*^*ires-CreERT2*^ (65% versus 50%). Second, we observe more corticotrophs when tracking cell fate with *Sox2*^*CreERT2*^ and more lactotrophs when using *Sox9*^*ires-CreERT2*^ (although the proportion of lactotrophs varied between animals; Table 1 and Table S1). As mentioned above, there is a small proportion of SOX2^+ve^;SOX9^−ve^ cells (Fauquier et al., 2008) and SOX9^+ve^; SOX2^−ve^ cells. The differences seen between the two fate-mapping strategies could therefore reflect the progeny of these single positive cells. Alternatively, or in addition, cell fate decisions may be affected by *Sox2* haploinsufficiency in *Sox2*^*CreERT2*^ animals (Kelberman et al., 2006).

To better characterize these differences, we first induced Cre activity in adult *Sox2*^*CreERT2/+*^*;R26R*^*EYFP/+*^ and *Sox9*^*ires-CreERT2/+*^;*R26R*^*EYFP/+*^ animals and analyzed progeny 1 week later, counting the proportion of EYFP^+ve^ cells that retain a progenitor identity. Of the EYFP^+ve^ cells, 96% remain SOX2;SOX9-double-positive (n = 4, SD = 1.3) in the latter, while only 77% (n = 3, SD = 6.2) did so in the former (^∗∗^p = 0.002). GH^+ve^ somatotrophs and ACTH^+ve^ corticotrophs were commonly seen among the differentiated (SOX2^−ve^;SOX9^−ve^) descendants of *Sox2*^*CreERT2*^ progenitors, whereas corticotrophs were very rare 1 week after induction when using *Sox9*^*ires-CreERT2*^ (Figure 6B). There were also more differentiated endocrine cells in *Sox2* heterozygous (*Sox9*^*iresGFP/+*^;*Sox2*^*βgeo/+*^ and *Sox2*^*EGFP/+*^) versus control (*Sox9*^*iresGFP/+*^) pituispheres (33%, 14.3%, and 4.17%, respectively). (*Sox2*^*βgeo*^ is described in Avilion et al., 2003.)

To determine whether *Sox2* haploinsufficiency renders cells more prone to differentiation, we performed lineage tracing analysis using *Sox9*^*ires-CreERT2/+*^;*R26R*^*EYFP/+*^*;Sox2*^*βgeo/+*^animals. Induction was performed shortly after birth and pituitaries were harvested at 4 weeks. There was only a slight difference in the proportion of cells retaining progenitor identity (EYFP;SOX9-double-positive) between *Sox2* haploinsufficient pituitaries (49.7%, SD = 7.2, n = 5) compared with *Sox9*^*ires-CreERT2/+*^;*R26R*^*EYFP/+*^ littermates (53.5%, SD = 6.3, n = 5). *Sox2* haploinsufficiency is therefore unlikely to explain most of the differences we observe. Stem cells in the CNS similarly express both SOX2 and SOX9, but in this case there is an obvious distinct role for the two proteins in subsequent progenitor lineages, with SOX2 remaining active in neuroblasts and SOX9 in glial cell progenitors (Scott et al., 2010). The use of the two Cre drivers to follow cell fates in the CNS would therefore lead to overlapping but quite skewed distributions, as it is not possible to limit Cre activity to just the stem cells. If similar differences between SOX2- and SOX9-expressing cells occur in the pituitary, these must be subtle or transient; therefore, the results most likely reflect the difference in efficiency between the two lineage tracing tools (see Discussion).

This is being explored further, but we decided to pursue adult lineage tracing only using *Sox9*^*ires-CreERT2*^ because, in contrast with *Sox2*^*CreERT2*^, both copies of the gene are present and also, *Sox2* haploinsufficiency affects pituitary development and function in both mice and humans (Kelberman et al., 2006). We had previously hypothesized that SOX9-positive cells may represent transit amplifying progenitors and this was based on the absence of SOX9 protein from early spheres (Fauquier et al., 2008). However, as we have shown here, SOX9-positive cells can form spheres (Figure 1) and this, along with the lineage tracing results, suggests that SOX9, like SOX2, is a bona fide marker for adult pituitary progenitors.

### SOX2- and SOX9-Positive Cells Maintain Their Identity in Adults

To assess the potential of adult progenitors, we induced Cre activity in 8-week-old *Sox9*^*ires-CreERT2/+*^;*R26R*^*EYFP/+*^ animals and harvested pituitaries between 6 and 12 months later. At these stages, there are very few EYFP^+ve^ cells and the vast majority are double positive for SOX2 and SOX9 (Figure 3M, Table 1, and Table S1). However, we were able to detect some differentiated progeny (Figure 3N long-term, Figure 6B short-term, and Figure S3). Moreover, some pituitary supporting cells, such as S100^+ve^ heterogenous folliculo-stellate cells, are among the descendants of the progenitors (Figure 3P). This was expected since we had previously shown that SOX2 and S100 can colocalize (Fauquier et al., 2008).

The fact that SOX9^+ve^ progenitors remain both SOX2- and SOX9-positive after long-term lineage labeling strongly suggests that both proteins are markers of stem cells and not transit amplifying progenitors (for review see Grompe, 2012). Short-term BrdU incorporation experiments show that the SOX2^+ve^;SOX9^+ve^ cells proliferate, although at a low rate, whereas very few SOX2-positive cells were found to be BrdU positive in label-retaining assays (Fauquier et al., 2008, and see below). Together, these results suggest that the stem cells self-renew, but differentiate rarely in the adult animal, at least when these are maintained in standard conditions. Therefore endocrine cell turnover in these circumstances may rely mostly on differentiated cell proliferation.

We then decided to analyze the regenerative potential of the SOX2^+ve^;SOX9^+ve^ cells in the gland in experimental situations with a greater demand for new endocrine cells.

### Pituitary Stem Cells Are Particularly Sensitive to the Mitogenic Effects of E2 In Vivo

**Figure 4:**
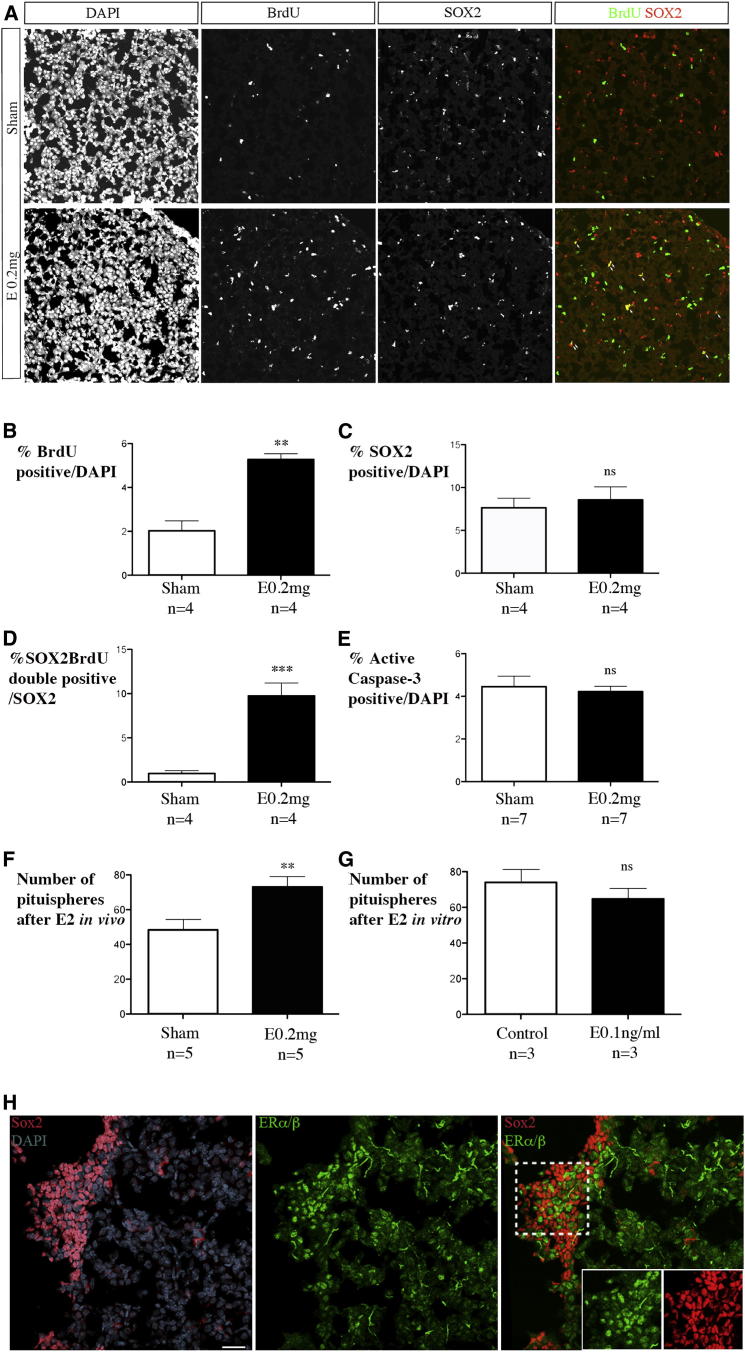
Estrogen Treatment in Males Induces Proliferation of Adult SOX2-Positive Cells In Vivo and Indirectly Induces Formation of More Pituispheres In Vitro E2 pellets (0.2 mg) were implanted in 8-week-old males. BrdU was then injected for 3 days and pituitaries were harvested. (A) Double immunofluorescence in a sham and E2-treated animals for BrdU and SOX2. There is more BrdU in the E2-treated sample and SOX2;BrdU-double-positive cells are numerous (arrows). (B) Percentage of BrdU-positive nuclei in sham versus treated animals. There is a significant increase of BrdU incorporation in E2-treated animals (p = 0.003). (C) The percentage of SOX2-positive cells in the anterior pituitary is not significantly affected by E2 treatment. (D) In contrast there is a significant increase in the percentage of SOX2;BrdU-double-positive/SOX2^+ve^ cells in E2-treated animals (p = 0.0003). (E) The percentage of caspase-3-positive cells is not affected. (F) In vivo E2 treatment increases significantly the number of pituispheres obtained after dissociation (p = 0.006). (G) In contrast, E2 treatment in vitro, after pituitary dissociation, does not affect the number of pituisphere formed. E2 effect on SOX2-positive cells is therefore likely to be indirect. (H) In agreement with this hypothesis, double immunofluorescence for SOX2 and ERα/β show almost exclusive expression patterns in the anterior pituitary. Scale bars: 50 μm. Data are presented as mean ± SEM.

E2 is a strong mitogen in the rodent pituitary, where it induces proliferation of existing endocrine cells and hormonally null cells and formation of new lactotrophs in males (Castrique et al., 2010; Nolan and Levy, 2006a, 2006b). These could be generated by division of existing lactotrophs and/or by differentiation from progenitors. In the latter case, maintenance of the pool of progenitors would require compensatory proliferation. We therefore looked at SOX2-positive cell proliferation after E2 treatment. Because there are relatively few SOX2-positive cells proliferating in the adult pituitary (Fauquier et al., 2008), we used BrdU as a marker and injected it daily for 3 days (Figure 4A).

Three days after the implantation of E2 pellets and daily BrdU injections, the percentage of BrdU-labeled cells/DAPI nuclei was more than doubled in treated animals (Figure 4B). We then compared the proportion of SOX2-positive cells in control and treated animals. Because we counted cells in fields encompassing the cleft, where SOX2-positive cells are the main cell type, and in the anterior lobe, the proportion of SOX2-positive cells in both groups is higher (about 8%, see Figure 4C) than observed in the whole pituitary (2%–3%, data not shown). However, this proportion is not significantly affected by E2 treatment (Figure 4C). In contrast, we observed ten times more SOX2;BrdU-double-positive cells in treated animals (9.75%) than controls (0.98%) (Figure 4D). The SOX2-positive population is therefore extremely sensitive to the proliferative effect of E2 within the anterior lobe population. The vast majority of these cells are SOX2;SOX9-double-positive: 94% of SOX2;BrdU-double-positive cells (n = 322 in three treated animals) were also SOX2/SOX9-double-positive. Such a proliferative effect might be expected to increase the total proportion of pituitary cells that are SOX2 positive, and indeed this is slightly elevated in E2-treated animals (+ 0.92%), but this is not statistically significant (we would expect, as we see here, a less than 1% increase).

Alternatively the effect on the proportion of SOX2-positive cells may be slight because once they have divided, a proportion of the daughter cells may stop expressing the protein, and differentiate or die. It has indeed been suggested that nascent pituitary cells are particularly susceptible to apoptosis (Nolan and Levy, 2006a). Moreover it has been shown that E2 can elicit an apoptotic response in the pituitary (for review see Seilicovich, 2010). We therefore quantified apoptosis by performing active caspase-3 staining, but we did not observe any effect (Figure 4E).

Therefore, SOX2/SOX9-positive cells proliferate in response to E2 treatment and are particularly sensitive to its mitogenic effect. This is in agreement with their proposed function as progenitors.

### E2 Effects on Pituitary Stem Cells Are Likely to Be Indirect

An increase in SOX2-positive cell proliferation in vivo should result in the formation of more spheres in vitro. To test this hypothesis, we dissociated pituitaries 3 days after E2 pellet implantation and seeded them at the same density from treated and control animals. We obtained a similar number of cells after dissociation from treated versus sham-operated animals, suggesting that the proliferative effect of E2 is not yet measurable. In contrast, when we counted spheres after 1 week of culture, there were significantly more spheres in cultures from treated animals (Figure 4F, p = 0.0055). To test whether the effects of E2 on the SOX2-positive cells are direct or indirect, we added it in vitro on freshly dissociated pituitary cells. In contrast with the previous experiment, in vitro E2 treatment did not affect pituisphere numbers (Figure 4G, p = 0.38), suggesting that the effect of E2 on SOX2-positive cells is indirect.

We then looked at the expression of the E2 receptors (ERs), finding that ERα and ERβ are widely expressed in the anterior lobe, as previously described (González et al., 2008). However, ER and SOX2 staining were mutually exclusive (Figure 4H), which is in agreement with an indirect effect of E2 on sphere-forming ability and on SOX2-positive cell proliferation.

### Pituitary Stem Cells Proliferate after Ablation of a Pituitary Target Organ

Pituitary target organ ablation, more specifically gonadectomy and adrenalectomy, induces a transient mitotic wave in the pituitary followed by new hormonal cells of the appropriate type to regulate the now ablated organ (gonadotrophs and corticotrophs, respectively) (Nolan and Levy, 2006b; Ibrahim et al., 1986). However, cell division is mainly observed in nonendocrine cells. Moreover, ablation of both gonads and adrenals does not result in an added mitotic effect, suggesting that the same cell population is stimulated by each treatment. This suggests that a population of undifferentiated cells, or progenitors, proliferate in response to organ ablation and later differentiate (Nolan and Levy, 2006b).

**Figure 5:**
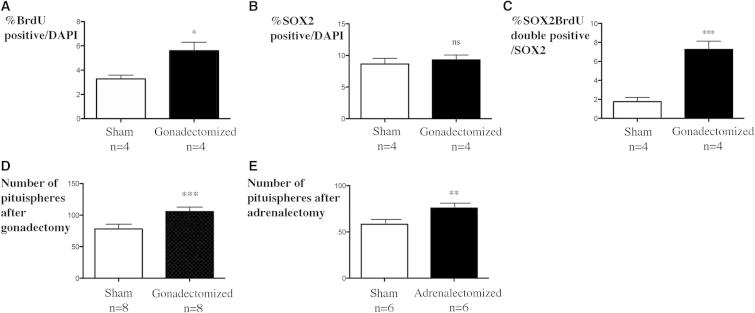
Pituitary Target Organ Ablation Induces Proliferation of Adult SOX2-Positive Cells In Vivo and Formation of More Pituispheres In Vitro Gonadectomies (A–D) and adrenalectomies (E) were performed in 8-week-old males. BrdU was then injected for 5 days and pituitaries were harvested (A–D). (A) Percentage of BrdU-positive nuclei in sham versus gonadectomized animals. There is a significant increase of BrdU incorporation in gonadectomized animals (p = 0.02). (B) The percentage of SOX2-positive cells in the anterior pituitary is not significantly affected by the ablation. (C) In contrast there is a significant increase in the percentage of BrdU;SOX2-double-positive/SOX2^+ve^ cells in gonadectomized animals (p = 0.001). Pituitaries from gonadectomized (D, p = 0.0006) or adrenalectomized (E, p = 0.005) animals give rise to significantly more spheres in vitro than their sham counterparts. Data are presented as mean ± SEM.

To ask if these cells include those that are SOX2 positive, we performed gonadectomies on young males and quantified proliferation after five daily injections of BrdU. As previously described (Nolan and Levy, 2006b), we found a significant increase in BrdU incorporation/DAPI in gonadectomized versus sham-operated animals (Figure 5A), albeit a more modest one than that induced by E2. As with the latter, there was a slight, but nonsignificant, increase in the percentage of SOX2-positive cells (Figure 5B). In contrast, there are four times more SOX2;BrdU-double-positive cells/SOX2-positive cells in gonadectomized compared to sham-operated animals (Figure 5C). Apoptosis, as assessed by active caspase-3 staining, was not affected by gonadectomies (data not shown). SOX2-positive cells are therefore included in the nonendocrine population that proliferates in response to organ ablation, in agreement with their progenitor function.

In order to better characterize this proliferative effect, we performed sphere assay experiments after gonadectomies and also adrenalectomies. Five days after surgery, pituitaries from sham-operated and operated animals were dissociated and cells were plated in sphere-forming conditions. We observed a significant increase in sphere formation after 1 week of culture after both gonadectomies (Figure 5D) and adrenalectomies (Figure 5E).

These data support a progenitor function for the SOX2;SOX9-double-positive cells. However, the increase in the percentage of SOX2;BrdU-double-positive cells does not by itself account for the global increase of BrdU incorporation, here and also after E2 treatment. After gonadectomies, 12.3% of the BrdU-positive cells are SOX2;BrdU-double positive compared to 4.5% in sham-operated animals. Therefore, while significantly accounting for the increase in BrdU incorporation, the SOX2^+ve^ population is not the only one proliferating in response to organ ablation. The same is true for the E2 experiment; however, it is well known that E2 broadly stimulates proliferation of pituitary cells (Castrique et al., 2010; Nolan and Levy, 2009). Gonadotrophs and corticotrophs have also been shown to proliferate after target organ ablation, although at a rate much lower than that of the SOX2-positive cells (our observations and Nolan and Levy, 2006b). It is possible that other classes of differentiated cells are stimulated by E2 and organ ablation. However, the immediate differentiating progeny of the stem cells, which will be SOX2^−ve^;SOX9^−ve^, could also account for a significant proportion of BrdU-labeled cells: an additional 12% if they do not divide at all, but all of them if they each divide just three times.

### Pituitary Stem Cells Give Rise to New Endocrine Cells after Target Organ Ablation and E2 Treatment

We next decided to use an organ ablation protocol in conjunction with lineage tracing in order to examine the differentiation potential of the SOX2^+ve^, SOX9^+ve^ progenitors in the adult. We chose adrenalectomies versus gonadectomies because new corticotrophs appear rapidly within 1 week after surgery, whereas the effects of castration on gonadotroph cell number occur later (Nolan and Levy, 2006b; Ibrahim et al., 1986).

**Figure 6:**
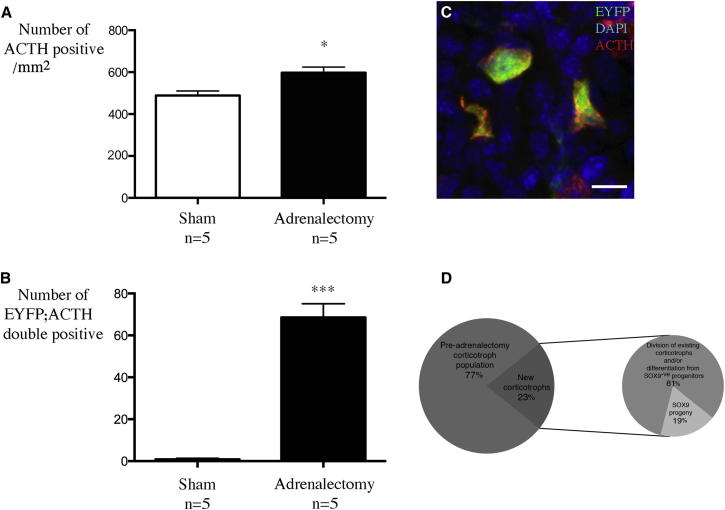
Pituitary Target Organ Ablation Induces Differentiation of Adult SOX9-Positive Stem Cells Adrenalectomies were performed 24 hr after the last tamoxifen administration (5 mg/25 g/day for 5 days). Pituitaries were harvested 1 week later. (A) ACTH-positive corticotrophs were counted on two sections/animal and the surface area of the sections was measured. We observed significantly more corticotrophs after adrenal ablation (p = 0.023). (B) We almost exclusively observe EYFP;ACTH-double-positive corticotrophs after adrenalectomies in *Sox9*^*ires-CreERT2/+*^*;R26R*^*EYFP/+*^ animals (adrenalectomy n = 5, 2 females and 3 males, sham-operated n = 5, 3 females and 2 males, p = 0.0005, with no difference found between males and females). (C) Double immunofluorescence illustrating the differentiation of EYFP^+ve^ SOX9 progenitors in ACTH^+ve^ corticotrophs in a *Sox9*^*ires-CreERT2/+*^*;R26R*^*EYFP/+*^ animal after adrenalectomy. Scale bar: 10 μm. (D) Proportion of new corticotrophs generated from SOX9^+ve^ progenitors. We first assessed the efficiency of SOX9-IRES-CreERT2 by counting the number of EYFP;SOX9-double-positive cells 48 hr after 5 days of tamoxifen treatment. Eighteen percent (SD = 3.2, n = 3) of SOX9^+ve^ cells were EYFP^+ve^. We then measured the surface area of all the sections where we counted ACTH;EYFP-double-positive cells in adrenalectomized animals (n = 5). We corrected this number so that it represented 100% and not just the 18% of cells generated from SOX9^+ve^ cells. This amounted to 21 corticotrophs/mm^2^, representing 19% of the new corticotrophs. Data are presented as mean ± SEM. See also Figure S4 and Table S1.

We treated 8-week-old *Sox9*^*ires-CreERT2/+*^;*R26R*^*EYFP/+*^ animals with tamoxifen for 5 consecutive days and performed adrenalectomies the following day. Pituitaries were harvested 6 days after surgery. We counted 23% more ACTH^+ve^ cells/surface area in adrenalectomized versus sham-operated samples (Figure 6A and Figure S4; both groups are tamoxifen treated), in agreement with previous studies (Nolan and Levy, 2006b; Subburaju and Aguilera, 2007). The majority of EYFP^+ve^ cells still retain SOX9 expression (data not shown), but when we counted EYFP;ACTH-double-positive cells, we observed a significant increase (Figure 6B, p = 0.0001) in the number of double-positive cells in adrenalectomized versus sham-operated animals (Figure 6B). To ensure that this effect was specific, we examined thyrotrophs, because these should not be affected by adrenalectomies. No EYFP;TSH-double-positive cells were observed (n = 2, in one sham and one adrenalectomized animal).

We then estimated the proportion of new corticotrophs, generated after adrenalectomies, which originate from differentiation of SOX9^+ve^ progenitors. We find that SOX9^+ve^ stem cells generate 21 corticotrophs/mm^2^, representing 19% of the newly generated corticotrophs (Figure 6D and Table S1). The remaining 81% are presumed to arise from division of existing corticotrophs (10% of the corticotrophs dividing once would be sufficient) and/or differentiation of SOX9^−ve^ committed progenitors.

Finally, we performed lineage tracing experiments after E2 treatment in *Sox9*^*ires-CreERT2/+*^*;R26R*^*EYFP/+*^ males. E2 effects on endocrine cell proliferation and numbers in males are relatively nonspecific within the different endocrine populations, with maybe a slightly higher sensitivity of lactotrophs (Castrique et al., 2010; A. Levy, personal communication) but we observed, in responding animals, prominent differentiation of the stem cells in the main anterior lobe endocrine population, GH^+ve^ somatotrophs (Figure S4).

In conclusion, SOX2- or SOX9-expressing adult pituitary stem cells can differentiate in response to pharmacological E2 treatment and, more importantly, to physiological demand, where they make a significant contribution to the number of new cells generated after target organ ablation.

## Discussion

In this study, we first demonstrated that SOX2^+ve^, and also SOX9^+ve^ cells, are the pituisphere-forming cells. We then showed, using genetic lineage tracing tools, that both embryonic and adult SOX2- and SOX9-positive cells give rise to pituitary endocrine cells, proving that they are progenitors. We previously hypothesized that SOX2^+ve^;SOX9^−ve^ cells represented stem cells while SOX2;SOX9-double-positive cells were likely to be shorter lived, more committed transit amplifying progenitors (Fauquier et al., 2008). In this present study, we show that, in fact, SOX9^+ve^ cells maintained their identity in long-term lineage tracing experiments. The vast majority of these cells are also SOX2^+ve^, and therefore SOX2;SOX9-double-positive cells, or at least many of them, represent adult stem cells (for review see Grompe, 2012). Furthermore, to explore the regenerative potential of these adult stem cells, we analyzed their proliferative response to two different protocols, E2 administration in males (Nolan and Levy, 2009) and target organ ablation (Nolan and Levy, 2006b), both suggested to stimulate proliferation of pituitary progenitors and generation of new endocrine cells (for review see Rizzoti, 2010). We have shown that both treatments dramatically increase proliferation of the SOX2;SOX9-double-positive cells and, in conjunction with genetic lineage tracing, we demonstrate that they can give rise to new endocrine cells of the appropriate type in response to target organ ablation, while E2 treatment in males induced differentiation predominantly into somatotrophs.

In the embryo, in agreement with endocrine cell birth-dating experiments (Davis et al., 2011), SOX2^+ve^ cells give rise to more hormone cells before 14.5 dpc, at which stage SOX9 is upregulated. SOX9 expression is therefore associated with a decreased differentiation rate. This suggests that SOX9 expression correlates with a switch of SOX2^+ve^ cell identity from a highly proliferative differentiative progenitor to a more quiescent stem cell.

Postnatally, the tamoxifen effect on gonadotroph cell numbers prevents us from making any conclusion about the normal physiological participation of progenitors in this phase of pituitary growth. They can clearly give rise to all endocrine lineages, so they are recruited to build the mature gland. However, the proportion of progenitors giving rise to gonadotrophs (about 35%) seems quite large in view of the slight increase in LH^+ve^ cell numbers (×1.2). Moreover, in adults, where tamoxifen does not have such an impact, stem cells mainly retain their identity, except when an additional stimulus, such as adrenalectomy, instructs them to differentiate. Therefore postnatal pituitary stem cells are mainly mobilized in specific or exceptional situations to generate new endocrine cells.

SOX2-CreERT2 and SOX9-IRES-CreERT2 postnatal lineage tracing results suggest that there are differences between both cell lineages despite the fact that more than 95% of the cells are double positive. In addition, we suspect that the difference in efficiency between both tools may affect lineage tracing results. With a less efficient tool, such as *Sox9*^*ires-CreERT2*^, we see far fewer recombined cells and this could (in proportion) favor the presence, in the EYFP^+ve^ population, of more long-lived stem cells than shorter-lived differentiated progeny.

Hormone deficiencies, congenital or acquired, can be managed by replacement or substitution therapies. However physiological secretion patterns cannot be mimicked by these treatments and it would be better to restore missing endocrine cells (for review see Castinetti et al., 2011). Here we have been able to stimulate proliferation and differentiation of stem cells into new somatotrophs after E2 treatment in males and, in response to adrenal ablation, into corticotrophs, within 1 week after surgery. More precisely, after adrenalectomies, differentiation from adult progenitors provided a significant proportion (19%) of the new corticotrophs. It is now important to investigate the physiological mechanisms instructing the stem cells to differentiate and integrate into the appropriate hormonal cell network (for review see Mollard et al., 2012), to be able to manipulate these in regenerative medicine, or, for example, to control aspects of reproductive function.

In conclusion, we have been able to show that in the pituitary, an organ where cell turnover is low and differentiated endocrine cells can proliferate, adult progenitors can be mobilized and made to differentiate toward a specific cell fate. The target organ ablation model we have used here will now allow us to dissect the mechanisms underlining this stem cell mobilization, in order to potentially direct new endocrine cell generation, particularly in pathological situations.

## Experimental Procedures

### Ethics Statement

All experiments carried out on mice were approved under the UK Animal (scientific procedures) Act (project license 80/2405).

### Mice

*Sox9*^*ires-CreERT2*/+^ (Furuyama et al., 2011) and *Sox2*^*CreERT2/+*^ (Arnold et al., 2011) mice were bred with *R26R*^*EYFP/EYFP*^ (Srinivas et al., 2001) mice for lineage tracing studies. Cre activity was induced by 4-OHT treatments in both pregnant females (0.1 to 0.15 mg/g/day) for embryo analysis and in P0/P2 pups (1 mg) and by tamoxifen treatment in adult (5 mg/25 g/day for 3 to 5 consecutive days). CBA/Ca × C57BL/10 F1 hybrids, 8–11 weeks old, were used for estrogen administration and organ ablation experiments. For each experiment (comprising three experimental and three sham operated animals), animals of the same age were used. For estrogen treatment, 0.2 mg (as pellets releasing 9.5 μg/day, β-oestradiol 3-benzoate, Innovative Research of America) was implanted subcutaneously under anesthesia. For gonadectomies and adrenalectomies, bilateral ablation was performed under anesthesia. BrdU (100 μg/g body weight) was injected daily starting the day after implantation/surgery. For lineage tracing after adrenalectomies, tamoxifen was administrated for 3 to 5 consecutive days at 5 mg/25 g/day and surgery was performed on day 6. Pituitaries were harvested 1 week after surgery.

### Immunofluorescence

Pituitaries were harvested and fixed by intracardiac perfusion with 4% PFA. Immunofluorescence was performed on cryosections as described (Rizzoti et al., 2004) using a goat anti-SOX2 antibody (ISL, 1/500), a goat or rabbit anti-SOX9 antibody (R&D and a gift from F. Poulat, Montpellier, France, respectively, 1/300), a rat anti-BrdU antibody (Abcam, 1/200), a rabbit anti-estrogen receptor antibody (Novus, 1/100), a rat anti-GFP antibody (Nacalai Tesque, 1/1,000), rabbit anti-Pit-1 (a gift from S. Rhodes, Indianapolis), and rabbit anti-GH, PRL, LH, and ACTH antibodies (provided by A.F. Parlow, NHPP, Torrance, USA) followed by anti-goat, rabbit, or rat Alexa 555, 488, or 647 secondary antibodies (Molecular Probes).

### Cell Counting and Statistical Analysis

For lineage tracing analysis, EYFP-positive cells were counted on a minimum of 10 fields randomly chosen and encompassing the whole pituitary. For estrogen treatment and endocrinolectomy, a minimum of 3,000 DAPI-positive nuclei was counted per animal, on four different fields taken on different sections (two encompassing the cleft and two in the anterior lobe), using cell-counting software (Zhu et al., 2010). Within these, the numbers of SOX2-positive, BrdU-positive, and SOX2;BrdU-double-positive cells were counted blindly and manually. For lineage tracing after adrenalectomies, pituitaries were sectioned serially on five slides. The number of ACTH/EYFP-double-positive cells was counted on one slide/animal and the surface area was measured using Fiji software. ANOVA tests were performed for statistical analysis using NCSS software. Angular transformation was applied to compare percentages.

### Pituitary Dissociation for FACS and Sphere Assay

Pituitaries were dissociated as described (Castrique et al., 2010), subject to FACS for GFP with mice carrying *Sox2*^*EGFP*^ (Ellis et al., 2004) or *Sox9*^*iresGFP*^ alleles (Furuyama et al., 2011), and cultured in sphere-forming conditions (Fauquier et al., 2008). To assess sphere-forming efficiency after estrogen treatment or endocrinolectomy, pituitaries were harvested 3 and 6 days after surgery, respectively, and spheres were counted blindly and manually after 1 week in culture. To assess the effects of estrogen in vitro, E2 (β-estradiol, Sigma) was added daily (0.1 ng/ml) and spheres were counted after 1 week in culture.

## Figures and Tables

**Table 1:** Lineage Tracing Quantification

		Number of EYFP^+ve^ Cells Counted	EYFP;^∗^SOX (Percent)	EYFP;Pit-1 (Percent)	EYFP;ACTH (Percent)	
Embryonic lineage tracing	SOX2-CreERT2, induced at 11.5 and 12.5 dpc, harvested 18.5 dpc	2426, n = 3	16, SD = 1.7	55.4, SD = 9.6	9.3, SD = 2.9	
	SOX2-CreERT2, induced at 13.5 dpc, harvested 18.5 dpc	684, n = 4	37, SD = 10			
				EYFP;Pit-1-ACTH (Percent)		
	SOX9-CreERT2, induced at 11.5 and 12.5 dpc, harvested 18.5 dpc	677, n = 7	37, SD = 9.6	41.4, SD = 3.8		

				EYFP;LH (Percent)	EYFP;ACTH (Percent)	EYFP;PRL (Percent)
Postnatal lineage tracing	SOX2-CreERT2, induced at P0/P2, harvested at 4 weeks	4106, n = 4	35, SD = 3.2	39, SD = 5.6	3.2, SD = 0.7	0.9, SD = 1.2
	SOX2-CreERT2, induced at P0/P2, harvested at 8 weeks	987, n = 2		42, SD = 2.5	6.7, SD = 0.2	
	SOX9-CreERT2, induced at P0/P2, harvested at 4 weeks	1897, n = 8	49, SD = 8.4	34, SD = 4.3	0.9 (n = 1)	7, SD = 7.3

				EYFP;SOX9;SOX2^+ve^ (Percent) within EYFP;SOX9	EYFP;SOX9;SOX2^−ve^ (Percent) within EYFP;SOX9	EYFP;Pit-1 (Percent)
Adult lineage tracing	SOX9-CreERT2, induced at 8 weeks, harvested at 6 to 12 months	436, n = 5	87, SD = 9.8	99, SD = 0.2 (n = 2)	1.15, SD = 0.2 (n = 2)	2 (n = 1)

Double or triple immunofluorescence was performed for EYFP and progenitor or endocrine markers. EYFP^+ve^ cells were counted in an average of 10 fields taken across the entire pituitary for each staining. Percentage of EYFP;marker^+ve^/EYFP^+ve^ was calculated. Numbers are given when fewer than three pituitaries were used for a single staining. ^∗^SOX = SOX2 for *Sox2*^*CreERT2*^ samples and SOX9 for *Sox9*^*ires-CreERT2*^. See also Table S1.

## Supplemental Information

Document S1. Figures S1–S4 and Table S1

Table S1. Lineage Tracing Analysis Related to Figure 2, Figure 3, and Table 1 Double-positive EYFP;marker cells were counted on an average of 10 different fields in the anterior lobe (excluding the cleft) across the entire pituitary. (^∗^) denotes countings on triple immunofluorescences. To estimate the proportion of new corticotrophs, generated after adrenalectomies, which originate from differentiation of SOX9^+ve^ progenitors, we first evaluated the efficiency of SOX9-IRES-CreERT2 in the pituitary; we administrated tamoxifen for 5 consecutive days, as done prior to adrenalectomies, and harvested pituitaries 48 hr after the last treatment. We observed that 18% of SOX9^+ve^ cells were EYFP^+ve^ (n = 3 SD = 3.2). Therefore 1 in 5.6 SOX9^+ve^ cells is labeled following Cre induction. We had previously counted the number of extra corticotrophs generated after adrenalectomies and observe a 23% increase in the number of corticotrophs, or an extra 113 cells/mm^2^ (Figure 6A). To estimate the proportion of corticotrophs within these extra 113 cells that come from a SOX9^+ve^ progenitors, we corrected the number of ACTH;EYFP cells we counted so that this number reflected participation from the whole SOX9 population and not just the 18% labeled by the use of SOX9-IRES-CreERT2, so we multiplied this number by 5.6 (18 × 5.6 = 100). We then measured the surface area corresponding to all the sections that were examined for ACTH;EYFP double-positive cells/animal so that we could calculate the proportion of ACTH cells generated from SOX9^+ve^ progenitors/mm^2^. We find that SOX9^+ve^ stem cells generate 21 corticotrophs/mm^2^, representing 19% of the newly generated corticotrophs.
