# Supplementary figures and images for: Mobilized Adult Pituitary Stem Cells Contribute to Endocrine Regeneration in Response to Physiological Demand
Source: Cell Stem Cell. 2013 Oct 3;13(4):419–32. doi: 10.1016/j.stem.2013.07.006 (PMC3793864; doi:10.1016/j.stem.2013.07.006)

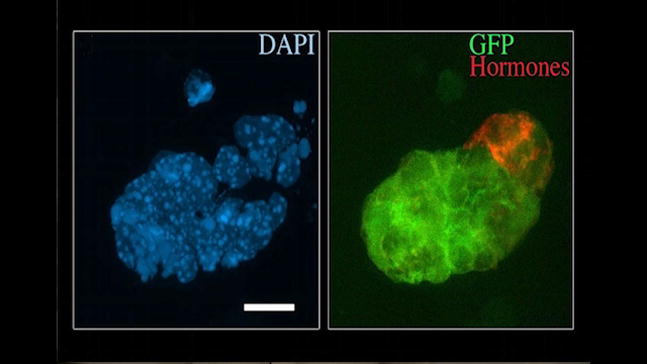

Supplement: Supplementary file 1 [file mmc3.jpg]
