# Supplementary material for: Mobilized Adult Pituitary Stem Cells Contribute to Endocrine Regeneration in Response to Physiological Demand
Source: Cell Stem Cell. 2013 Oct 3;13(4):419–32. doi: 10.1016/j.stem.2013.07.006 (PMC3793864; doi:10.1016/j.stem.2013.07.006)
Supplement: Document S1. Figures S1–S4 and Table S1 [file mmc1.pdf]

Figure S1

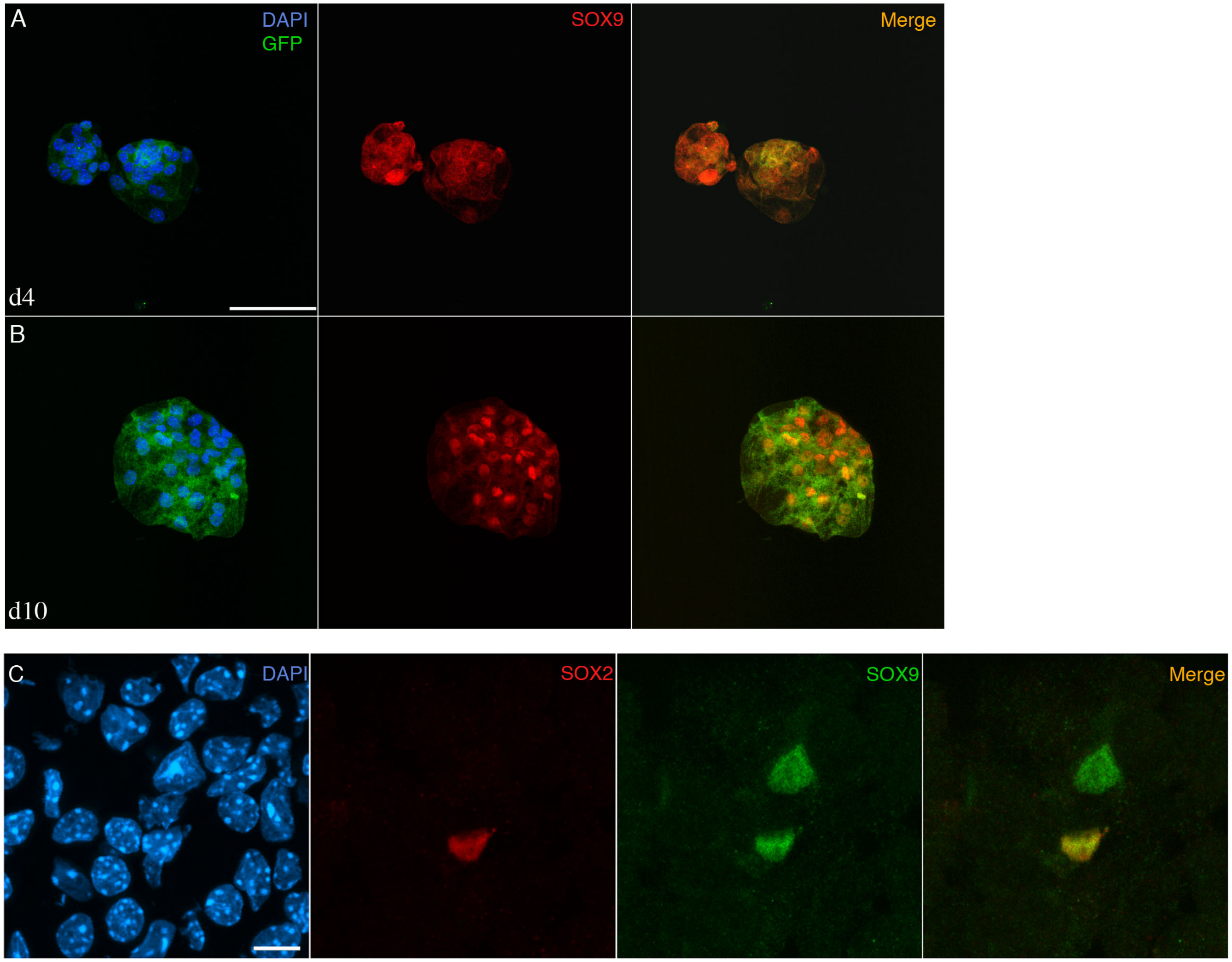

Figure S2

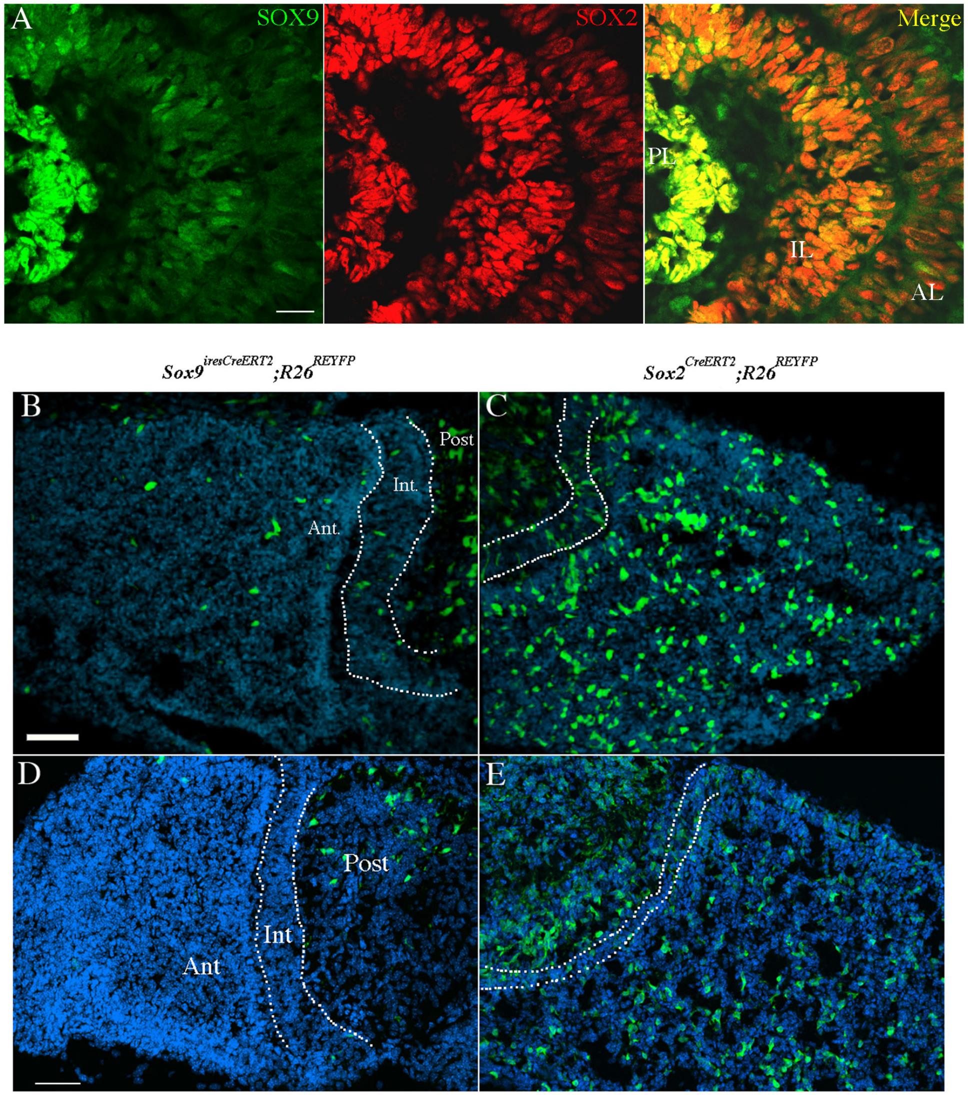
A
SOX9
SOX2
Merge
PL
IL
AL
Sox9
iresCreERT2
;R26
REYFP
Sox2
CreERT2
;R26
REYFP
B
Post
Int.
Ant.
C
D
Post
Int
Ant
E

Figure S3

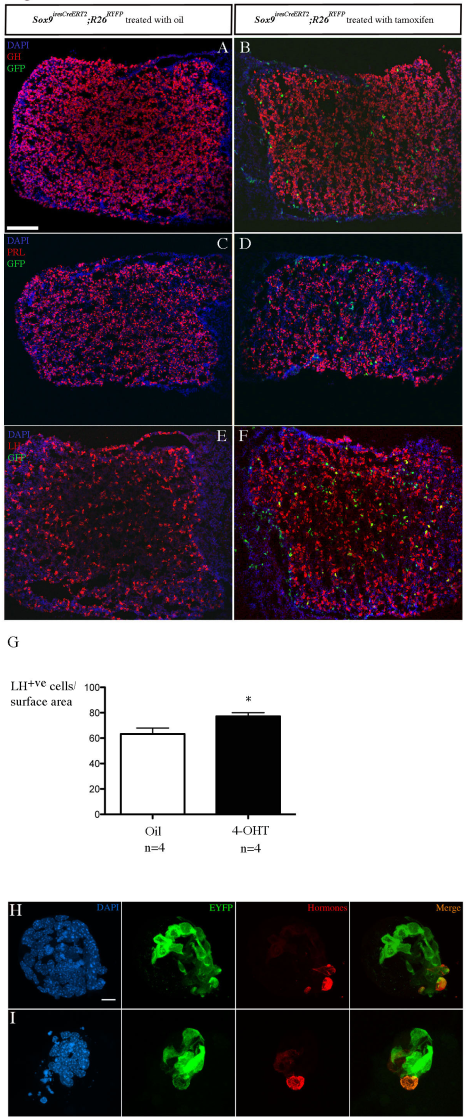

Sox9^iresCreERT2;R26^RYFP treated with oil
Sox9^iresCreERT2;R26^RYFP treated with tamoxifen
DAPI
GH
GFP
A
B
DAPI
PRL
GFP
C
D
DAPI
LH
GFP
E
F
G
LH+ve cells/ surface area
100
80
60
40
20
0
*
Oil n=4
4-OHT n=4
H
DAPI
EYFP
Hormones
Merge
I

Figure S4

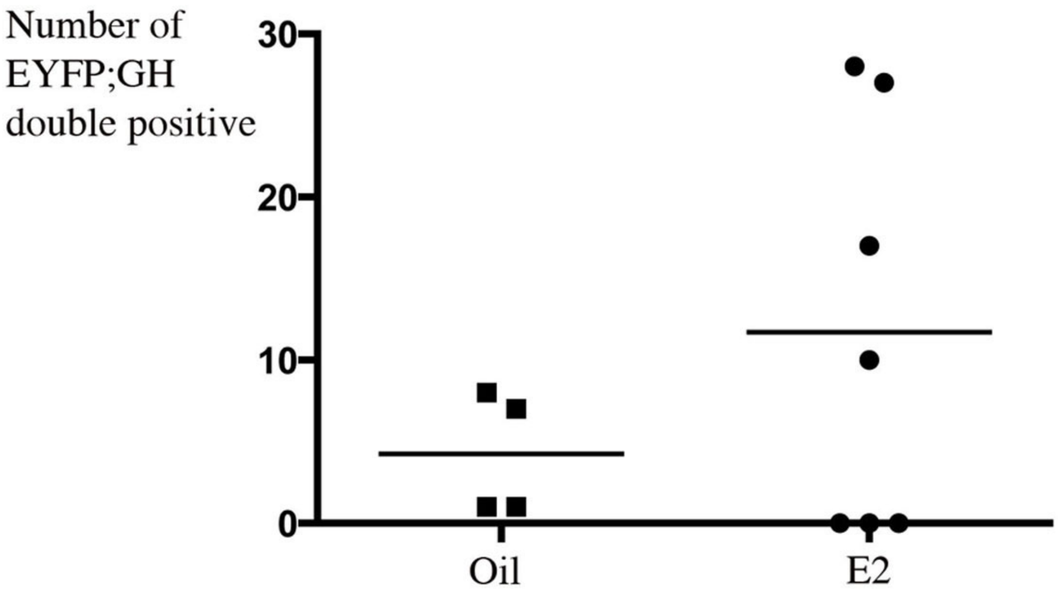

Supplemental Data.

Inventory:

Figure S1: SOX9 expression is down-regulated during early sphere formation related to Fig.1.

Figure S2: SOX9 expression is up-regulated later than SOX2 and there is a difference in efficiency between *Sox9*$^{ires\text{-}CreERT2}$ and *Sox2*^{CreERT2} related to Fig.2.

Figure S3: Early Tamoxifen treatment post-natally affects gonadotroph numbers and SOX9 and SOX2 positive cells give rise to endocrine cells other than gonadotrophs *in vitro* related to Fig.3.

Figure S4: SOX9 positive stem cells differentiate into somatotrophs in response to estrogen treatment in males related to Figure 6.

Table S1: Lineage tracing analysis related to Fig.2, 3 and Table 1.

Supplementary Figure Legends:

Figure S1: SOX9 expression is down-regulated during early sphere formation related to Fig.1.

A) Pituispheres were obtained from adult SOX9-IRES-GFP pituitaries. GFP; SOX9 double immunofluorescences show presence of GFP in early spheres, at day 4, while SOX9 protein is itself rarely seen. In contrast, at day 10, SOX9 is clearly up-regulated. D) Double immunofluorescence of an adult male anterior pituitary showing a SOX9^{+ve};SOX2^{-ve} cell. These represent 1.4% of the SOX9^{+ve} population in the anterior pituitary (n=1363 in 3 animals, sd0.5) while SOX2^{+ve};SOX9^{-ve} represent 1.9% of the SOX2^{+ve} population (n=1363 in 3 animals, sd0.3). Scale bar is and 50μm for A-B 10 μm for C.

Figure S2: SOX9 expression is up-regulated later than SOX2 and there is a difference in efficiency between *Sox9*$^{ires\text{-}CreERT2}$ and *Sox2*^{CreERT2} related to Fig.2.

A) SOX2; SOX9 double immunofluorescence on a 14.5dpc pituitary. This is the earliest stage when SOX9 is clearly up-regulated in the SOX2^{+ve} cells lining the cleft, particularly on the future intermediate lobe side.
Pregnant R26R^{EYFP} females from either *Sox9*$^{ires\text{-}CreERT2}$ (B;D) or *Sox2*^{CreERT2}(C; E) males were similarly treated with 4 OHT at 11.5 and 12.5dpc (B; C 0.1 to 0.15mg/g/day) or 13.5dpc (D; E) and pituitaries harvested at 18.5dpc. There is more recombination using SOX2-CreERT2, and tamoxifen treatments are reproducibly more efficient when administered at 11.5 and 12.5 than at 13.5 dpc. Scales bar are 10 µm for A and 55µm for B-E.

<u>Figure S3</u>: Early Tamoxifen treatment post-natally affects gonadotroph numbers and SOX9 and SOX2 positive cells give rise to endocrine cells other than gonadotrophs *in vitro* related to Fig.3.
A-F) *Sox9*$^{ires\text{-}CreERT2\ /+}$;*R26R*$^{EYFP/+}$ P0/P2 pups were injected with 1mg of 4 OHT (B; D; F) or an equivalent volume of corn oil (A; C; E) and pituitaries harvested 4 weeks later. Double immunofluorescence for EYFP and GH (A; B), PRL (C; D) and LH (E; F) were performed. EYFP is only observed when tamoxifen is administrated, demonstrating that Cre activity strictly depends on induction. There is an apparent increase in LH numbers in induced animals and EYFP; LH double positive cells are observed (E; F). Somatotrophs do not seem affected (A; B) while lactotroph numbers may be reduced by tamoxifen (C; D). Scale bar is 150 µm. G) LH^{+ve} cells were counted on at least 2 sections/animal and surface area measured to obtain the proportion of gonadotrophs. There is a significant increase in the proportion of gonadotrophs in 4-OHT treated animals ($p=0.044$, $n=4$, 2 males and 2 females we did not observe any difference between sexes), this is likely to explain the bias observed in lineage tracing experiments where the majority of SOX2 and SOX9 differentiated progeny are LH positive. Data are presented as mean +/- SEM.
In order to definitively prove that SOX2 and SOX9^{+ve} cells give rise to endocrine cells *in vitro*, we performed lineage tracing experiments on *Sox2*$^{CreERT2/+}$; *R26R*$^{EYFP/+}$ and *Sox9*$^{ires\text{-}CreERT2/+}$; *R26R*$^{EYFP/+}$ spheres. H) *Sox9*$^{ires\text{-}CreERT2/+}$;*R26R*$^{EYFP/+}$ spheres were generated from 3 to 4 week-old animals. 4-OHT was added *in vitro* and spheres differentiated, as described (Fauquier et al., 2008). Double-immunofluorescence for EYFP and GH shows that SOX9 positive cells can give rise to somatotrophs. This confirms that the high proportion of gonadotrophs observed in the SOX9 progeny was a consequence of the tamoxifen treatments and not a reflection of the progenitor potential. I) *Sox2*$^{CreERT2/+}$;*R26R*$^{EYFP/+}$ spheres from 4 week-old

animals were similarly treated and differentiated. Double immunofluorescence against EYFP and pituitary hormones show differentiation from a $SOX2^{+ve}$ cell. Scale bar is 10 μm.

Figure S4: SOX9 positive stem cells differentiate into somatotrophs in response to estrogen treatment in males related to Figure 6.

Tamoxifen was administrated for 2 days (5mg/25g/day) in adult $Sox9^{ires\text{-}CreERT2/+}$;$R26R^{EYFP/+}$ males (n=11 8 weeks to 6 month-old). 3 days later (to allow for elimination of the estrogen receptor modulator tamoxifen), estrogen injections were started (n=4, oil as control, n=7, E2), as described (Castrique et al, 2010). Estrogen is known to increase prolactin secretion and has also been suggested to increase the proportion of lactotrophs but is in fact stimulating proliferation relatively a-specifically in the anterior pituitary (Castrique et al, 2010). We however mainly, and sometimes exclusively, observe somatotrophs in the progeny of adult pituitary stem cells after E2 treatment in responding males. Note that some animals (3 out of 7, this didn't correlate with age) did not respond to estrogen (we did not observe any EYFP;GH double positive cells), maybe because of the earlier administration of tamoxifen.

Table S1: Lineage tracing analysis related to Fig.2, 3 and Table 1.

Double positive EYFP;marker cells were counted on an average of 10 different fields in the anterior lobe (excluding the cleft) across the entire pituitary. * denotes countings on triple immunofluorescences.

To estimate the proportion of new corticotrophs, generated after adrenalectomies, which originate from differentiation of $SOX9^{+ve}$ progenitors, we first evaluated the efficiency of SOX9-IRES-CreERT2 in the pituitary; we administrated tamoxifen for five consecutives days, as done prior to adrenalectomies, and harvested pituitaries 48h00 after the last treatment. We observed that 18% of $SOX9^{+ve}$ cells were $EYFP^{+ve}$ (n=3 sd3.2). Therefore 1 in 5.6 $SOX9^{+ve}$ cells is labelled following Cre induction. We had previously counted the number of extra corticotrophs generated after adrenalectomies, and observe a 23% increase in the number of corticotrophs, or an extra 113 cells/mm^2 (Fig. 6A). To estimate the proportion of corticotrophs, within these extra 113 cells that come from a $SOX9^{+ve}$ progenitors, we corrected the number of ACTH;EYFP cells we counted so

that this number reflected participation from the whole SOX9 population and not just the 18% labelled by the use of SOX9-IRES-CreERT2, so we multiplied this number by 5.6 (18 x 5.6=100). We then measured the surface area corresponding to all the sections that were examined for ACTH;EYFP double positive cells/animal so that we could calculate the proportion of ACTH cells generated from $SOX9^{+ve}$ progenitors/mm^2. We find that $SOX9^{+ve}$ stem cells generate 21 corticotrophs/mm^2, representing 19% of the newly generated corticotrophs.
